# The Study of Naphthoquinones and Their Complexes with DNA by Using Raman Spectroscopy and Surface Enhanced Raman Spectroscopy: New Insight into Interactions of DNA with Plant Secondary Metabolites

**DOI:** 10.1155/2014/461393

**Published:** 2014-06-22

**Authors:** Veronika Vaverkova, Oldrich Vrana, Vojtech Adam, Tomas Pekarek, Josef Jampilek, Petr Babula

**Affiliations:** ^1^Department of Natural Drugs, Faculty of Pharmacy, University of Veterinary and Pharmaceutical Sciences, Palackeho 1-3, 612 42 Brno, Czech Republic; ^2^Institute of Biophysics, Academy of Sciences of the Czech Republic, Kralovopolska 135, C612 65 Brno, Czech Republic; ^3^Department of Chemistry and Biochemistry, Faculty of Agronomy, Mendel University in Brno, Zemedelska 1, 613 00 Brno, Czech Republic; ^4^Zentiva, k.s., Development Department, U Kabelovny 130, 102 37 Praha 10, Czech Republic; ^5^Department of Chemical Drugs, Faculty of Pharmacy, University of Veterinary and Pharmaceutical Sciences, Palackeho 1-3, 612 42 Brno, Czech Republic

## Abstract

Naphthoquinones represent the group of plant secondary metabolites with cytotoxic properties based on their ability to generate reactive oxygen species and interfere with the processes of cell respiration. Due to this fact, the possible cytotoxic mechanisms on cellular and subcellular levels are investigated intensively. There are many targets of cytotoxic action on the cellular level; however, DNA is a critical target of many cytotoxic compounds. Due to the cytotoxic properties of naphthoquinones, it is necessary to study the processes of naphthoquinones, DNA interactions (1,4-naphthoquinone, binapthoquinone, juglone, lawsone, plumbagin), especially by using modern analytical techniques. In our work, the Raman spectroscopy was used to determine the possible binding sites of the naphthoquinones on the DNA and to characterize the bond of naphthoquinone to DNA. Experimental data reveals the relationships between the perturbations of structure-sensitive Raman bands and the types of the naphthoquinones involved. The modification of DNA by the studied naphthoquinones leads to the nonspecific interaction, which causes the transition of B-DNA into A-DNA conformation. The change of the B-conformation of DNA for all measured DNA modified by naphthoquinones except plumbagin is obvious.

## 1. Introduction

Naphthoquinones are naturally widespread secondary metabolites, the products of some actinomycetes (*Streptomyces* Waksman and Henrici), fungi (*Fusarium* Link,* Marasmius* Fr., and* Verticillium* Nees), lichens, algae, and plants. The most important naphthoquinones-containing plants belong to the group of phylogenetically heterogeneous plant families Avicenniaceae Endl. ex Schnizl., Balsaminaceae DC., Bignoniaceae Juss., Boraginaceae Juss., Droseraceae Salisb., Ebenaceae Gurke, Juglandaceae A. Rich. ex Kunth, Nepenthaceae Dum., and Plumbaginaceae Juss. [1–4]. The chemical structure of the monomeric naphthoquinones is based on the bicyclic system—naphthalene skeleton substituted in the positions C_(1)_ and C_(4)_ (1,4-naphthoquinones) or C_(1)_ and C_(2)_ (1,2-naphthoquinones). There are dimeric and trimeric naphthoquinones, which evidence significant cytotoxic properties on different tumour cell lines including prostate androgen-dependent cell lines, and breast tumour cell lines [5, 6]. Naphthoquinones have many physiological roles; the most important ubiquitous naphthoquinones are vitamins of the K group—phylloquinone and menaquinone [7–9]. Plenty of naphthoquinones are important phytoalexins, so they play an important role in ecological relationships [10]. Naphthoquinones have found many possibilities for use—antimicrobial, antifungal, antiviral, and antiprotozoal properties have been identified. In traditional medicines, particularly in Asia (China) and South America, plants with naphthoquinones have a wide range of application, especially in the treatment of various types of cancer [2]. The cytotoxic properties of the naphthoquinones are in the focus of interest of many scientists. They are predominantly based on the ability of naphthoquinones to generate reactive oxygen species (ROS), which are involved in many cellular processes and serve as signal molecules in apoptosis/programmed cell death and are partially responsible for cytotoxic properties demonstrated in many* in vitro* tumour cell line systems [11–14]. The ability of naphthoquinones to interfere with the respiratory chain poses the second possible cytotoxic mechanism [15–17]. DNA is the critical target of the activity of many compounds including plant secondary metabolites. There is a large group of natural origin compounds with interesting cytotoxic properties. Their cytotoxic effect is based on their interactions with DNA, especially on the possibility of forming various types of covalent adducts with DNA. The most important compounds, whose effect is based on DNA interaction, are anthracyclines, cytotoxic antibiotics, and platinum-based cytostatics [18–21].

DNA-metal and DNA-metal based complexes interactions and their effect on DNA structure are already known [22–29]. Cisplatin as an important and frequently used anticancer drug forms covalent bonds with nucleophilic sites on guanine residues present in DNA. As a cisplatin is a bifunctional agent, it is able to bind to one (monofunctional adduct) or two (intrastrand crosslinks, interstrand crosslinks) places in a DNA chain [30–32]. All of these adducts characteristically distort the DNA conformation [33, 34]. The main problem in the curing process with many anticancer complexes such as cisplatin is the reduced sensitivity of the tumour towards this drug when the drug is repeated in the treatment. A medical treatment is connected with many inadvisable effects [35, 36]. Therefore, new structures with reduced toxicity, often of natural origin, are intensively searched. Due to significant cytotoxic properties of the selected naphthoquinones (see Figure S1 in Supplementary Material available online at http://dx.doi.org/10.1155/2014/461393), we decided to study the naphthoquinones-DNA interactions as a possible mechanism of their cytotoxicity. In recent years, naphthoquinone derivatives have been intensively studied in connection with their cytotoxic and antibacterial effects and subsequently possible usage in cancer treatment [37–41]. Despite the above-mentioned mechanisms of naphthoquinones cytotoxicity, their interactions with DNA have not yet been established. Due to this fact, we focused on the investigation of naphthoquinones-DNA interactions in our work. The identification of the binding sites of naphthoquinones on DNA and the determination of how the binding may affect secondary and tertiary structures is still lacking. Some naphthoquinones have been identified as potent inhibitors of topoisomerases I and II; this activity may contribute to their cytotoxic effect [2, 37, 42]. In addition, newly synthesized complexes of lawsone and metal ions are recently investigated as cytotoxic compounds with the cytotoxic effect based on the DNA interactions [43]. The vibrational spectra [44, 45], optical absorption and fluorescence emission spectra [46, 47], and SERS studies [44, 45, 48–51] of some naphthoquinone derivatives have also been reported. However, the naphthoquinones-DNA interactions can play the crucial role in their cytotoxic effect. The vibrational spectroscopy (Raman spectroscopy) can help making unambiguous identification of vibrational modes of biological molecular systems [24, 32]. We used Raman spectroscopy to assess the binding sites of the naphthoquinones to DNA and to characterize and compare the perturbations of DNA after its modification by naphthoquinones in our work. We used Raman spectroscopy and SERS in the analysis of DNA modified by the selected naphthoquinones: 1,4-naphthoquinone, binaphthoquinone, juglone, lawsone, and plumbagin (Figure S1). The spectra obtained were analyzed and characteristic spectral changes were described with the aim of determining interactions between selected naphthoquinones and DNA and analyzing DNA structural changes under naphthoquinones-DNA interactions. In addition, the linear dichroism spectroscopy was used to verify these results.

## 2. Materials and Methods

### 2.1. Chemicals, Material and pH Measurements

1,4-Naphthoquinone, binaphthoquinone [2,2-bi(3-hydroxy-1,4-naphthoquinone], juglone [5-hydroxy-1,4-naphthoquinone], lawsone [2-hydroxy-1,4-naphthoquinone], and plumbagin [5-hydroxy-2-methy-1,4-naphthoquinone] as well as all other chemicals of ACS purity unless noted otherwise were purchased from Sigma Aldrich Chemical Corp. (Sigma-Aldrich, USA). Deionised water underwent demineralization by reverse osmosis by using an instrument Aqua Osmotic 02 (Aqua Osmotic, Tisnov, Czech Republic) and was subsequently purified by using a Millipore RG (Millipore Corp., USA, 18 M*Ώ*)—MiliQ water. The pH value was measured by using the WTW inoLab pH meter (Weilheim, Germany).

### 2.2. Preparation of DNA

DNA was isolated from the calf thymus in accordance with the published work of Brabec et al. [33]. DNA was dissolved in 0.5 M NaCl and was then sonicated for 20 minutes by using a Sonicator 3000 (Giltron, USA) and subsequently analyzed. The first step of denatured DNA analysis was the electrophoresis by using the source Standard Power Pack P25 by (Biometra, Germany). The gel was photographed after the backlighting the gel by UV on a transilluminator TVX-20M (312 nm) (AlysLabware, Switzerland). The next step was the polarography on an analyzer EG&G PARC Model 384B (LabX, Canada). The concentration of DNA was measured on a Beckman DU 7400 (Pall Gelman, Germany). For the dialysis, a Spectra/Por 12-14000 (Cole-Palmer, USA) was used. The lyophilization was performed on a Labconco 4.5 (LabX, Canada). To remove the proteins, which cause the fluorescence of DNA in Raman spectroscopy, phenol and chloroform extractions were used. The final DNA concentration was 2 × 10^−1^ M.

Naphthoquinones were dissolved in a volume of 3 mL in the purified Milli-Q water. The final concentrations of the selected naphthoquinones were 1,4-naphthoquinone—3.2 × 10^−1^ M, binaphthoquinone—3.1 × 10^−1^ M, juglone—2.9 × 10^−1^ M, lawsone—3.2 × 10^−1^ M, and plumbagin—2.8 × 10^−1^ M.

### 2.3. Raman Spectroscopy

Spectra were recorded on the Raman spectrometer, Jobin-Yvon T64000 (Horiba, France).

### 2.4. Surface-Enhanced Raman Spectroscopy

The preparation of Ag colloid: component A—5.15 mg of hydroxylamine hydrochloride was added to 5 mL of redistilled water, and component B—6.00 mg of sodium hydroxide was also added to 5 mL of redistilled water. Both these components were mixed and immediately added to the solution of silver nitrate (8.48 mg silver nitrate in 45 mL of redistilled water). The colloid was measured using UV spectroscopy and then compared with previous studies [33, 52, 53]. The silver (Ag) colloid complex (of naphthoquinones) was prepared by mixing equal volumes of the solution of naphthoquinones or DNA with the Ag colloid to obtain the final concentration of 5 × 10^−7^ M or 5 × 10^−6^ M. The final concentration of the colloid was 9 × 10^−4^ M. The spectra of the prepared samples were measured immediately after mixing the naphthoquinone or DNA with Ag colloid at 25°C. Samples (10 *μ*L) were sealed in a glass capillary. The measurement of the whole spectrum was divided into two parts and each part consisted of several accumulations (16 × 2 s).

### 2.5. Preparation of DNA-Naphthoquinone Complexes

To prepare each naphthoquinone-DNA complex, DNA was incubated with the naphthoquinones in the solution of 0.01 M NaClO_4_ at 37°C in the dark for 24 hours.

### 2.6. Instrumentation

The complexes (naphthoquinones + DNA) were dissolved to a final concentration of 30 mg/mL in 0.1 M NaCl (pH 7.0). Aliquots (4 *μ*L) of such prepared complexes were sealed in a glass capillary Kimax 34507 (Kimble products, USA). Spectra of these aliquots were excited at 488 nm using an argon ion laser (Innova 90C Fred, Coherent, USA). The radiant power at the sample was set to 100 mW and it was monitored by a Broadband Power Meter (MellesGriotte, USA). Measurements were performed at 25°C in back scattering geometry. Rayleigh line was removed from the scattered light by a Kaiser Optical notch filter. The Raman signal was detected in a single mode using 1200 lines/nm grating, entrance slit of 0.1 mm wide, and a spectrum One CCD3000 LN2 cooled detector. Spectrometer and detector were controlled by JobinYvonLabSpec software version 3.03t (Horiba, France).

The Raman spectra of individual naphthoquinones used for complexation were measured prior to the colloid spectral analysis. Spectra of these molecules were acquired directly in the solid phase in the same instrumental settings as mentioned above. The laser power was set to 10 mW.

### 2.7. Data Processing

Some spectra were disturbed by a significant fluorescent background. It was approximated by the polynomial function and subtracted by using “baseline correction” functionality of the LabSpec software. For the normalization of the different spectra, the Raman band near 1092 cm^−1^ assigned to the phosphate group vibration was used as an internal intensity standard. The intensity of this band remains the same regardless of the A-DNA or B-DNA conformation [54]. The processing of the data measured was carried out by using the LabSpec software (Horiba, France). Spectra were further converted to Omnic 6.2 (Thermo, USA) format.

### 2.8. Linear Dichroism (LD)

LD was used to confirm the intercalation of the naphthoquinones to DNA. LD spectra were recorded by using a flow Cuvette cell in a Jasco J-720 spectropolarimeter adapted for LD measurements (Jasco Analytical Instruments, Japan). Long molecules such as DNA can be orientated in the flow Cuvette cell. The flow cell consists of a fixed outer cylinder and a rotating solid quartz inner cylinder, separated by a gap of 0.5 mm, giving a total path length of 1 mm [55]. LD spectra of ctDNA modified by the selected naphthoquinones were recorded at 25°C in 10 mM NaClO_4_.

## 3. Results

### 3.1. Control of DNA Denaturation

DNA was sonicated and the possible denaturation of DNA was checked (see Supplementary Material).

### 3.2. Raman Spectra of DNA Modified by Selected Naphthoquinones (Solid Phase, Solutions)

The overview of the selected naphthoquinones' Raman spectra is given in Figure S3. These spectra are used further for the evaluation of the Raman spectra of the colloid-DNA complexes in the following chapters.

### 3.3. The Analysis of DNA Modified by 1,4-Naphthoquinone

**Figure 1:**
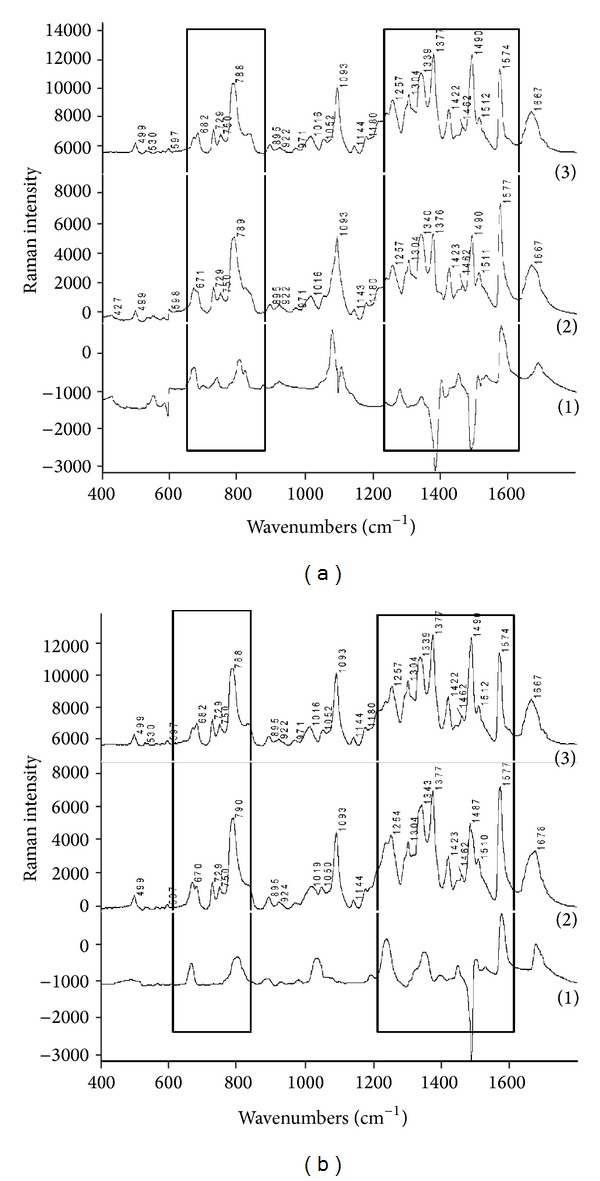
Raman spectra (a): (1) difference Raman spectrum of DNA and 1,4-naphthoquinone (spectrum 2-spectrum 3), (2) Raman spectrum of DNA and 1,4-naphthoquinone, and (3) control ctDNA (the areas with the differences in spectra are in the border), (b): (1) difference Raman spectrum of DNA and binaphthoquinone (spectrum 2-spectrum 3), (2) Raman spectrum of DNA and binaphthoquinone, and (3) control ctDNA (the areas with the differences in spectra are in the border).

We used the main bands in the spectra for the comparison of the changes in the cases of the modified DNA by selected naphthoquinones. These bands are used for description of interaction in cases of different compounds [1, 31, 45, 56–59]. Only the most important spectral features are discussed in the following chapters; particular bands assignment is present in Table 2. The bands near 670 and 683 cm^−1^ are a marker of C3′-*endo*/*anti*-conformation in case of A-form DNA (A-DNA). The intensity of the band near 670 cm^−1^ decreases together with the conformation of the sugar part of deoxyriboguanosine conversion from C2′-*endo*/*anti*-conformation to C3′-*endo* conformation [31, 60–62]. The ratio of bands' intensities 683/669 cm^−1^ > 1 indicates that the DNA modified by the complex is in the B-conformation. Higher intensity of the band at 669 cm^−1^, the higher the portion of the modified DNA in the A-form. Thus, the final ratio of the bands' intensities 683/669 cm^−1^ < 1 proves the A-conformation of the modified DNA by 1,4-naphthoquinone (Figure 1(a)). There is not an intensive band at 834 cm^−1^ in DNA modified by 1,4-naphthoquinone and this fact supports the assumption of the transition from B-DNA to A-DNA. The increase of the band at 804 cm^−1^ in DNA modified by 1,4-naphthoquinone indicates the local structural changes and it is the next evidence of A-form presence. The band at 788 cm^−1^ in the DNA is a combination band of cytosine, thymine, and the phosphodiester band. This band is therefore very intensive also in the modified DNA. The bands of DNA (modified and unmodified) in the region 800–1050 cm^−1^ correspond to the vibrations of the sugar-phosphate backbone skeleton. The intensity change of the band near 1078 cm^−1^ is influenced by the contribution of 1,4-naphthoquinone (Figure 1(a)). The band at 1340 cm^−1^ corresponds to the vibration of adenine and guanine. Changes of the intensity of this band indicate changes in the pairing between GC and AT bases. In the DNA modified by 1,4-naphthoquinone stacking interaction was interrupted and 1,4-naphthoquinone was fastened onto adenine in DNA structure. The band at 1377 cm^−1^ in the spectrum of DNA modified by 1,4-naphthoquinone is influenced by the thymine vibration. Due to changes of adenine-thymine pairing the intensity of this band is changed in the modified DNA spectrum compared to native DNA. The band 1504 cm^−1^ (in B-DNA is located in the area of 1511 cm^−1^) in DNA modified by 1,4-naphthoquinone is assigned to the vibration of imidazole ring of adenine and the increasing intensity of this band indicates a partial modification of adenine and guanine. The band in DNA modified by 1,4-naphthoquinone near 1574 cm^−1^ corresponds to the overlapping contributions from the dG and dA. This band is located at 1577 cm^−1^, in the spectrum of B-DNA. The increasing intensity of this band in the case of DNA modification by 1,4-naphthoquinone results in disruption of hydrogen bonds in GC and AT pairs. This fact is related with the predenaturation and denaturation changes in DNA modified by 1,4-naphthoquinone. The band at 1688 cm^−1^ corresponds to the carbonyl group stretching vibration of the (C=O) group of the thymine and its width is influenced by the hydrogen bonds of thymine and adenine.

### 3.4. Analysis of DNA Modified by Binaphthoquinone

The ratio of intensities at 683/670 cm^−1^ is 0.7 which indicates the transition from the B-conformation in A-conformation (Table 3) in the spectrum of DNA modified by binaphthoquinone (Figure 1(b)) like in the case of DNA modified by 1,4-naphthoquinone. Furthermore, changes in the DNA modified by binaphthoquinone in the intensity of the band at 804 cm^−1^ are observed. This is in concordance with changes from the B-conformation (834 cm^−1^) into the A-conformation (807 cm^−1^) and this change coincides with an increase in the intensity of band 670 cm^−1^. The band at 1339 cm^−1^ in the spectrum of native DNA is a combination band of adenine, guanine, and thymine. In the spectrum of DNA modified by binaphthoquinone this band indicates high distorsions in AT and GC base pairs and large local denatruration was obvious and the large local denaturation was obvious.

### 3.5. Analysis of DNA Modified by Juglone

**Figure 2:**
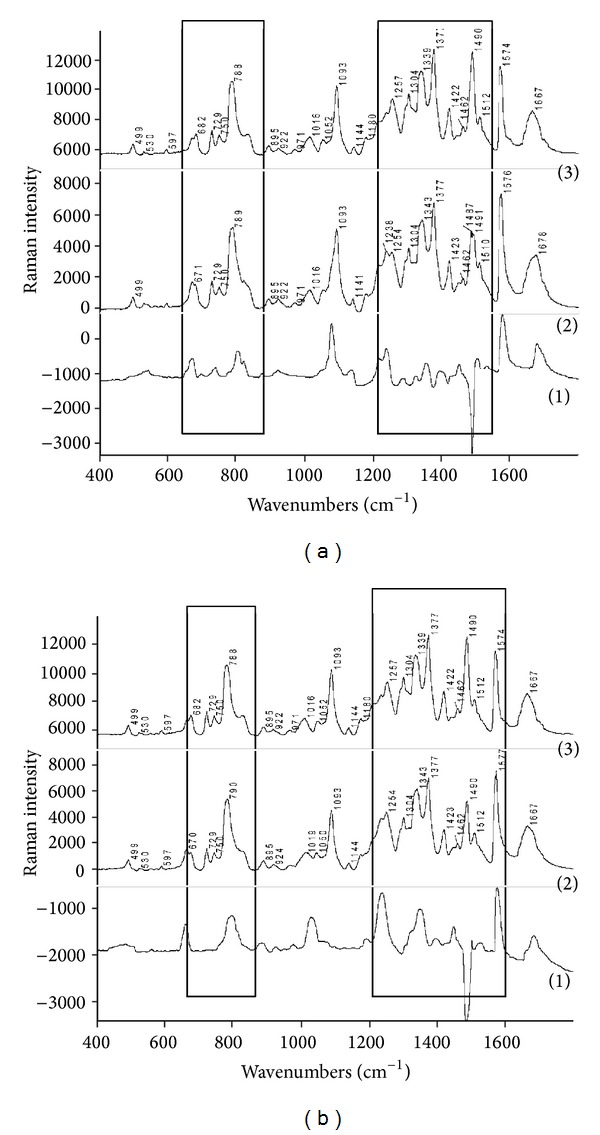
Raman spectra (a): (1) difference Raman spectrum of DNA and juglone (spectrum 2-spectrum 3), (2) Raman spectrum of DNA and juglone, and (3) control ctDNA (the areas with the differences in spectra are in the border), (b): (1) difference Raman spectrum of DNA and lawsone (spectrum 2-spectrum 3), (2) Raman spectrum of DNA and lawsone, and (3) control ctDNA (the areas with the differences in spectra are in the border).

DNA modified by juglone was changed in the areas around 670 cm^−1^ and 683 cm^−1^ compared to the native DNA. The ratio of the bands' intensities 683/670 cm^−1^ is 0.9 (Table 3), which indicates the transition from the B-conformation in A-conformation (Figure 2(a)) as it was in the case of DNA modified by 1,4-naphthoquinone and binaphthoquinone. This fact is also supported by the highest increasing intensity of the band at 807 cm^−1^ in the case of DNA modified by juglone. The band at 1237 cm^−1^ represents the vibrations of cytosine and thymine in the native DNA, and its increase in the case of DNA modified by juglone corresponds to the local denaturation. In DNA modified by juglone the increase of intensity near 1343 cm^−1^ is related to the changes in the deoxyribonucleoside conformation in the GC and AT pairs, which is significant for the local denaturation (as well as the band at 1237 cm^−1^). In the difference Raman spectrum of DNA modified by juglone the increase of the band at 1503 cm^−1^ is clearly observable; this intensity change is related to the hydrogen bond of adenine and thymine. This means that the AT hydrogen bonds have been broken and juglone was fastened to the adenine in the DNA structure.

### 3.6. Analysis of DNA Modified by Lawsone

From the difference Raman spectrum of DNA modified by lawsone it is clear that there are changes in the areas near 670 cm^−1^ and near 683 cm^−1^ (Figure 2(b)). In the DNA modified by lawsone the intensity of the band at 670 cm^−1^ increased more than the intensity of the band near 683 cm^−1^. The ratio of bands' intensities at 683/670 cm^−1^ is 0.7 (Table 3), which indicates the transition from the B-conformation of native DNA to A-conformation of the modified DNA, similarly to DNA modified 1,4-naphthoquinone, binaphthoquinone, and juglone. We also identified changes in the spectra of DNA modified by lawsone near 807 cm^−1^, which also indicates changes from B-conformation (834 cm^−1^) in the A-conformation (807 cm^−1^) and this change coincides with an increase in the intensity of band at 670 cm^−1^. The band at 1343 cm^−1^ is slightly shifted in DNA modified by lawsone comparing to native DNA and it corresponds to the vibrations of adenine, guanine, and thymine. There have been high distortions in AT and GC base pairs and therefore there was a large local denaturation in AT and GC base pairs. In DNA modified by lawsone the band at 1577 cm^−1^ increased its intensity by more than 18% than in the case of DNA modified by 1,4-naphthoquinone and binaphthoquinone. This change indicates much more noticeable disruption of the hydrogen bonds in GC and TA pairs (similar to the band at 1338 cm^−1^) and greater predenaturation and denaturation effects than if it was in the case of DNA modified by 1,4-naphthoquinone and binaphthoquinone.

### 3.7. Analysis of DNA Modified by Plumbagin

**Figure 3:**
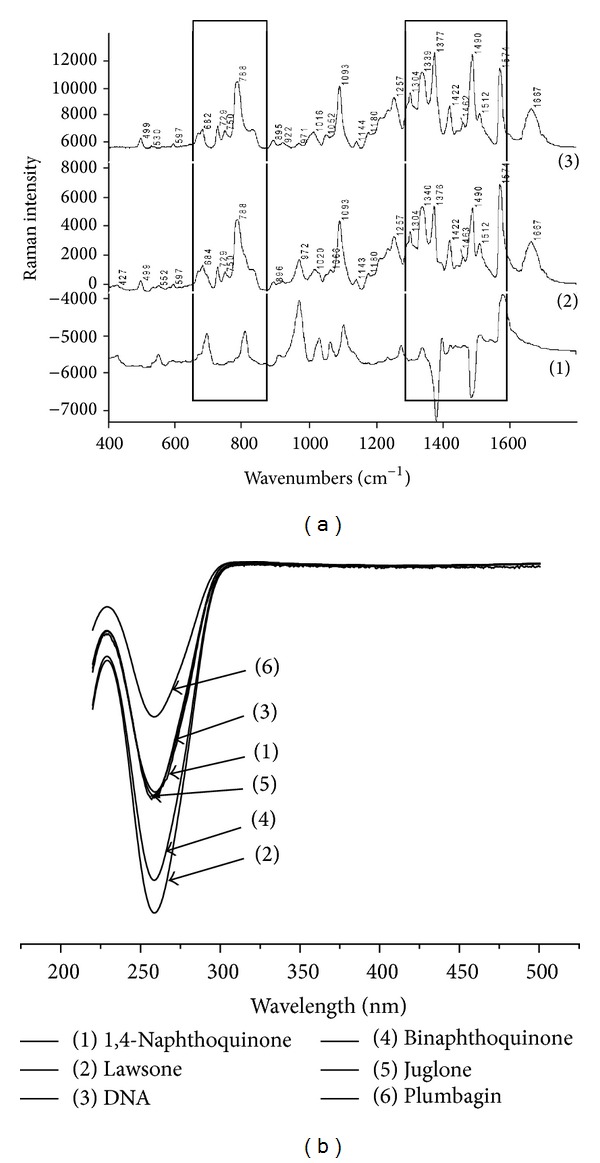
(a): Raman spectra (1) difference Raman spectrum of DNA and plumbagin (spectrum 2-spectrum 3), (2) Raman spectrum of DNA and plumbagin, and (3) control ctDNA (the areas with the differences in spectra are in the border), (b) linear dichroism (LD) spectra of calf thymus DNA modified by (1) 1,4-naphthoquinone, (2) lawsone, (3) control, nonmodified DNA, (4) binaphthoquinone, (5) juglone, and (6) plumbagin.

From the Raman spectra of DNA modified by plumbagin it is obvious that modified DNA was not transformed in the areas around 670 cm^−1^ and 683 cm^−1^ (Figure 3(a)). The ratio of the bands' intensities 683/670 cm^−1^ is 1.3 (Table 3), which does not indicate the transition from the B-conformation DNA in A-conformation. In DNA modified by plumbagin the band at 1236 cm^−1^ corresponds to vibrations of cytosine and thymine, and in this area the local denaturation is obvious. There were some denaturation changes in the DNA structure when modified by plumbagin but these changes do not inevitably indicate that plumbagin was intercalated into DNA.

### 3.8. Results Summary of All Measured Raman Spectra of DNA Modified by Selected Naphthoquinones (Solid Phase, Solutions)

The results obtained by Raman spectroscopy were compared with previously published studies where the DNA interactions with some substances were studied [31, 54, 60, 62–74]. The increase (decrease) of the selected bands intensity in the Raman spectrum of DNA after modification by the studied naphthoquinones is summarized in Table 1. All the changes of the Raman band positions and intensities after the modification of DNA by the selected naphthoquinones are introduced in Table 2. Table 3 shows changes in DNA conformation after its modification by naphthoquinones. It is obvious that after modification of DNA by all complexes, DNA was changed from B-conformation to A-conformation except of DNA modified by plumbagin.

### 3.9. Summary Results of All the Measured SERS Spectra of DNA Modified by Selected Naphthoquinones

The SERS method was used to confirm the hypothesis that substances bind to DNA and to confirm which main bases are modified after this binding to DNA (see Supplementary Material).

### 3.10. Linear Dichroism Spectroscopy

Long molecules such as DNA show a signal in the LD spectra (if it is oriented specifically). Small unbound molecules show no signal. The intensity of the LD signal of DNA modified by binaphthoquinone and lawsone had been decreased in the area of 260 nm. A smaller decrease in this intensity of LD signal was observed in the case of DNA modified by juglone and 1,4-naphthoquinone and the lowest decrease in intensity of LD signal in the case of DNA modified by plumbagin. This decreasing intensity of the peak 260 nm means the intercalation of the studied naphthoquinones into DNA. The decreases of the intensities of the LD band (220–300 nm) confirm that DNA modified by all studied naphthoquinones is shifted from B-DNA to A-DNA [55]. The largest intercalation mode of naphthoquinone to DNA is observed in the case of DNA modified by binaphthoquinone and lawsone, where the decrease of LD band at 260 nm is the most significant and the molecule of DNA by this modification is the most rigid. The results measured on the LD spectroscopy confirm the assumption that all the selected naphthoquinones intercalate into DNA in the range of lawsone > binaphthoquinone > juglone > 1,4-naphthoquinone > plumbagin (Figure 3(b)).

## 4. Discussion

It was necessary to reflect on the dramatic changes and to compare these changes in the cases of the modified DNA by 1,4-naphthoquinone, binaphthoquinone, juglone, lawsone, and plumbagin. We use the main bands in the spectra for these comparisons. These bands are widely used for description of interaction in cases of different compounds with DNA [1, 31, 45, 56–59]. The bands at 670 cm^−1^ and 683 cm^−1^ are markers of A or B-conformation in DNA. These two peaks are associated with the vibration of deoxynucleosides dT (mainly 670 ± 3 cm^−1^) and dG (683 cm^−1^). These markers are used in the studies of interactions between proteins and DNA [75]. Changes in the band near 670 cm^−1^ indicate the changes in A-/B-DNA conformation (see Table 3). However, they cannot be interpreted independently but in the comparison of the changes of the intensities of the band at 683 cm^−1^. The band near 670 cm^−1^ is a marker of C3′-*endo*/*anti*-conformation in case of A-form DNA, and in the B-form it is primarily due to dT. Decrease of the intensity of this peak is observed when conformation of the sugar moiety of deoxyriboguanosine is converted from C2′-*endo*/*anti*-conformation to C3′-*endo* conformation [31, 60, 61]. It is important to note that Raman band near 660–685 cm^−1^ is also sensitive to the sugar pucker and glycosyl torsion of deoxyguanosine nucleoside residues [31, 60, 61]. The signals in the area 800–1100 cm^−1^ are markers of changes in backbone geometry and DNA secondary structure [54]. All the selected naphthoquinones are responsible for the transition of DNA from B to A conformation except the DNA modified by plumbagin. The band at 807 ± 6 cm^−1^ is in the A-conformation DNA (A-form DNA phosphodiester marker) counterpart of the intensive band 834 cm^−1^ in B-conformation (phosphodiester B-form marker) [54]. The band 807 cm^−1^ is thus the marker of A-conformation (if the nucleoside conformation does not remain in the C2′-*endo* conformation) and its intensity increased the most for DNA modified by binaphthoquinone and plumbagin. By contrast, the intensity increased less for DNA modified by 1,4-naphthoquinone, juglone, and lawsone The band at 834 cm^−1^ is located in the area of 800–1000 cm^−1^. Bands in this area are sensitive to changes in the secondary structure of DNA and the changes in the sugar-phosphate backbone skeleton. The band at 834 cm^−1^ is one of the characteristic markers of B-DNA conformation and the corresponding vibration of the phosphodiester links [45, 76–78]. In the A-conformation of DNA, this band is shifted to lower values to the area from 807 to 811 cm^−1^, as already was mentioned. In the spectrum of modified DNA by 1,4-naphthoquinone and plumbagin, this band is slightly lower than in the comparison in the spectrum of the DNA modified by binaphthoquinone, juglone, and lawsone which is in good agreement with the data above-mentioned. The band at 1073 cm^−1^ is partly related to the vibration of the sugar-phosphate backbone (probably electrostatic interaction of the phosphate group) [79–82], but it especially corresponds with the vibrations to the contribution of the naphthoquinones measured in the solid phase—in the case of juglone and plumbagin. The area of band near 1237 cm^−1^ (1100–1400 cm^−1^) is in the spectrum of a very rich DNA. The changes in the area of 1200–1600 cm^−1^ correspond mostly to the changes of the vibration of purine and pyrimidine, so the bands in this area are associated with changes in the structure of the aromatic ring of purine and pyrimidine, which are sensitive to the binding of metal atoms to the aromatic [60, 65, 83]. Intensity of the band at 1237 cm^−1^, which is related to the vibration of thymine and cytosine, increased mostly for DNA modified by juglone (129% of control) and lawsone (144% of control). The significant increase of the intensity of the band results is the contribution of the cytosine and thymine vibration in DNA; it is associated with the bond of the studied naphthoquinones on the DNA and is a significant feature of the local denaturation. Band at 1237 cm^−1^ was not used only for description of* in vitro* systems, but also in systems represented by the cell lines [84]. The vibrational mode at 1338 cm^−1^ is assigned to the vibration of adenine and guanine and signified the local denaturation in AT and GC base pairs [85]. On the other hand, this band indicates formation of triple helices [86]. The band is sensitive to the changes in the electron structure of aromatic ring of purine and pyrimidine. In the case of DNA modification by 1,4-naphthoquinone there is a slight increase in the peak affected by 1,4-naphthoquinone contribution measured in the solid phase and this contribution is predominated by the conformation changes in GC and AT pairs. The significant increase in this peak was observed also for DNA modified by binaphthoquinone and lawsone. In contrast to this, only a small decrease of 1338 cm^−1^ was observed in the DNA modified by juglone. This result indicates the role of substitution of the naphthoquinone skeleton by hydroxyl groups. The bands at 1376 cm^−1^ and 1352 cm^−1^ confirm the local denaturation and they are related to the vibration of thymine, especially its methyl group [80, 87–89]. The band near 1376 cm^−1^ is linked in the case of DNA modified by binaphthoquinone, juglone, lawsone, and plumbagin with the contribution of these complexes measured in the solid phase. It can be explained that in the case of DNA modified by these naphthoquinones the band intensity increased not only by the contribution of the local denaturation but also by the contribution of these complexes themselves. The change of the band at 1490 cm^−1^ is assigned to the vibration of guanine and especially adenine [62, 90, 91]. Changes in the difference Raman spectra of this signal were observed for the DNA modified by all studied naphthoquinones. When there is the obvious decrease in the difference Raman spectrum in this area, it is related to the modification of N_(7)_ guanine. The N_(7)_ site of guanine is recognized as an important site of the identification of some metal interaction with DNA [92]. The size of the band at 1490 cm^−1^ decreases after electrophile binding agents to the N_(7)_. After the modification of DNA by selected naphthoquinones, this decrease is related with the guanine vibration not with the metal interaction with DNA.

The band at 1511 ± 4 cm^−1^ is the vibration mode of the imidazole's ring of adenine, particularly adenine stretching vibration [93]. This band intensity increased and it is slightly shifted in case of DNA modification by 1,4-naphthoquinone, binaphthoquinone, juglone, lawsone, and plumbagin. These changes indicate the significant disruption of the hydrogen bonds between adenine and thymine. Similar results are shown in the work of Iyandurai and Sarojini, who focused on the interactions between spermine and DNA [93]. Moreover, in the case of DNA modified by the selected naphthoquinones the band intensity at 1537 cm^−1^ increased. This is linked to the aromatic ring vibration of cytosine (in the B-conformation of DNA this band is in the area 1531 cm^−1^) and this change therefore well coincides with the changes from B-DNA to A-DNA. The characteristic point of the difference spectrum of DNA modified by all the naphthoquinones studied is the decrease and subsequent increase in the intensity at 1578 ± 4 cm^−1^ and 1586 ± 4 cm^−1^, respectively. Both of these bands are related to the vibration of adenine and guanine. The significant increase of the band intensity at 1578 ± 4 cm^−1^ can be interpreted as a pronounced disruption of the hydrogen bonds in GC and TA pairs. Similar results have been established for the investigation of alive and dead cells (MLE-12 cell line), where dead cells demonstrated significant increase of this peak [94]. This fact is related to the predenaturation and denaturation effect occurring in DNA modified by all measured naphthoquinones. The band at 1578 cm^−1^ increased the most in the case of DNA modified by binaphthoquinone. This change indicates very high disruption of the hydrogen bonds in GC and TA pairs and large predenaturation and denaturation effects in DNA modified by binaphthoquinone. The opposite results were demonstrated in the case of paclitaxel and 5-fluorouracil, where significant decrease of band near 1578 cm^−1^ was detectable [85, 95]. On the other hand, these two studies were carried out on the individual tumour cells. From the changes mentioned above it is clear that all the measured naphthoquinones except plumbagin transfer DNA from B-conformation to A-conformation. In the case of DNA modified by plumbagin, some local denaturation changes were observed; thus some interactions between DNA and plumbagin take place but it is not obvious if plumbagin intercalated into DNA. By using SERS and LD spectroscopy the interaction was confirmed. In the SERS spectra the changes of the spectra included changes in the intensities and in the positions of the bands too. This analysis demonstrates that naphthoquinones bind specifically to DNA and cause a specific distortion in the DNA.

## 5. Conclusions

All the studied DNA modifications by the selected naphthoquinones induced changes in the spectral part, which corresponds to the vibration of the sugar-phosphate backbone, in the areas of vibration of the bases, and also in the areas of vibration of each naphthoquinone. The modification of DNA by the studied naphthoquinones leads to the nonspecific interaction, which causes the distortion of DNA, from B-conformation of DNA to A-conformation. The change of the B-conformation of DNA for all measured DNA modified by naphthoquinones except plumbagin is obvious. It is also evident from the SERS spectra that the interaction between DNA and complexes takes places at the bases and not at the sugar-phosphate backbone. In the case of modification of the bases, the adenine and thymine were modified in all DNA modified by naphthoquinones. The area around 1200 ± 5 cm^−1^ was affected by the local denaturation. The area around 1600 ± 5 cm^−1^ exhibits no significant changes. For each DNA modified by all studied complexes the intensity of the band near 1277 cm^−1^ increased, which is affected by the vibration of adenine and guanine. It is obvious from these results that all naphthoquinones are bound to DNA except of plumbagin. In the case of plumbagin there were only some denaturation effects after the modification of DNA due to the methyl group in the naphthalene skeleton. The analysis of the difference spectra leads to the clear conclusions that bases have been modified, and these changes have predenaturation and denaturation character. Nevertheless, the results demonstrate that naphthoquinones bind specifically to DNA and cause a specific distortion. The cytotoxic activity of some naphthoquinones may be clarified in this way.

## Supplementary Material

Supplementary material provides access to essential data that do not appear in the main article. The supplementary material is important for the whole manucscript because it contains pictures of the selected naphthoquinones, control of DNA denaturation, Raman spectra of all measured naphthoquinones in the solid phase and SERS spectra of 1,4-naphthoquinone, binaphthoquinone, juglone, lawsone, and plumbagin.

## Figures and Tables

**Table 1:** The increase (decrease) of the bands in the Raman spectrum of DNA after the modification by the studied naphthoquinones. The changes are expressed in the percentages (∗the observation of Raman intensity of calf thymus DNA represents 100%; the change is counted separately for each band).

DNA-naphthoquinone	DNA∗	1,4-Naphthoquinone	Binaphthoquinone	Juglone	Lawsone	Plumbagin
670 cm^−1^ vibrations of thymine	100	181	191	190	186	95
683 cm^−1^ vibrations of guanine	100	112	101	118	101	120
834 cm^−1^ B-conformation, phosphodiester bond	100	96	98	98	98	99
1237 cm^−1^ vibrations of cytosine and thymine	100	85	147	129	144	102
1256 cm^−1^ vibrations of cytosine and thymine	100	81	116	95	113	100
1338 cm^−1^ vibrations of adenine and guanine	100	102	115	97	114	105
1376 cm^−1^ vibrations of thymine	100	78	109	101	107	86
1511 cm^−1^ vibrations of adenine	100	108	118	119	116	116
1578 cm^−1^ vibrations of adenine and guanine	100	125	126	123	125	123

**Table 2:** The changes of the Raman intensities after the modification of DNA by the selected naphthoquinones: w-weak, m-medium, s-strong, sh-shoulder, br-broad, as-asymmetric.

Peak (cm^−1^)	Assignment	1,4-Naphthoquinone	Binaphthoquinone	Juglone	Lawsone	Plumbagin
670	dT, dA	670 m	667 m	667 m	667 m	667 w
683	dG	—	—	—	—	—
729	A	—	—	—	—	—
750	dT	740 w	—	741 w	—	—
787	bk O-P-O str. + dT	805 m	804 m, br	807 m, br	807 m	807 m
834	bk O-P-O B-DNA	827 w	—	821 w	—	—
894	dr, C2′H_2_ rock	—	—	—	—	—
922	dr, ring str.	—	—	—	—	916 m
970	T C_6_H op-def, bk	—	—	—	—	—
1014	T CH_3_ rock	—	—	—	—	1018 sh
1054	bk C-O str.	1079 m-s	1038 m, br	1076 m-s	1039 m, br	1073 s, br
1093	POsym., str.	1110 w	—	—	—	—
1143	dT	—	—	1142 w	—	1147 m
1178	dT, dG, dC	—	—	—	—	1176 w
1217	dT, dA, dG	—	—	1215 sh	—	1216 m, as
1237	dT, dC	—	1239 m-s, br	1237 m	1240 m, br	—
1256	dC, dA, dT (dG)	—	—	—	—	—
1307	dA, dT	—	—	1281 w	1304 w	—
1338	dA, dG	—	—	1323 w	1322 sh	—
1376	T, CH_3_def	1377 s, 1398 w	1350 m, br	1359 m	1355 m, br	1384 w
1421	dr C5′H_2_def	—	—	—	—	—
1446		1448 w	1452 w	1448 w	1448 w	1442 w
1462		—	—	—	—	—
1490	G im. ring, dA, dT	1490 s, as	1490 s	1490 s	1490 s, as	1490 s
1511		1509 w	1502 w	1502 w	1503 w, sh	1507 m
1578	dG, dA	1574 s, as	1578 s, as	1578 s, as	1579 s, as	1576 s
1669	T, dG	1688 w	1688 m, as	1685 m, as	1689 w, br	1680 m-s, br, as

**Table 3:** The ratio of the size of the peak intensities at 683/670** **cm^−1^ with a peak intensity of 683** **cm^−1^.

DNA-naphthoquinone	683/670 cm^−1^
1,4-Naphthoquinone	0.8
Binaphthoquinone	0.7
Juglone	0.9
Lawsone	0.7
Plumbagin	1.3

## Abbreviations

A-DNA: Right-handed double helix, shorter more compact helical structure
B-DNA: Right-handed double helix
bp: Base pair
bk: Backbone
Cisplatina, cis DDP: cis-[(NH_3_)_2_Cl_2_Pt]
ct DNA: Thyme DNA
ds DNA: Double-strand DNA
ss DNA: Single-strand DNA
dA: Deoxyadenosine
dC: Deoxycytidine
dG: Deoxyguanosine
DNA: Deoxyribonucleic acid
DPP: Difference pulse polarography
dT: Deoxythymidine
LD: Linear dichroism
Wavenumber: (cm^−1^).
