# Supplementary material for: The Study of Naphthoquinones and Their Complexes with DNA by Using Raman Spectroscopy and Surface Enhanced Raman Spectroscopy: New Insight into Interactions of DNA with Plant Secondary Metabolites
Source: Biomed Res Int. 2014 Jun 22;2014:461393. doi: 10.1155/2014/461393 (PMC4090563; doi:10.1155/2014/461393)
Supplement: Supplementary file 1 — Supplementary material provides access to essential data that do not appear in the main article. The supplementary material is important for the whole manucscript because it contains pictures of the selected naphthoquinones, control of DNA denaturation, Raman spectra of all measured naphthoquinones in the solid phase and SERS spectra of 1,4-naphthoquinone, binaphthoquinone, juglone, lawsone, and plumbagin. [file 461393.f1.docx]

**Figure S1.** Naphthoquinones: a) 1,4 – naphthoquinone, b) binaphthoquinone, c) juglone, d) lawsone, e) plumbagin

**Control of DNA denaturation**

It is known that native double-stranded DNA molecule (ds) provides a small peak on the DPP curve (called peak II) in the area of -1.38 V ([Havran and others 2004](#_ENREF_35)). The slight increase in temperature called pre-denatured temperature indicates the increase of the peak II intensity. As a result of an intervention to the areas of the single-strand DNA (ss) in the sample (e.g. by increasing the temperature above the denaturation temperature), another peak (called peak III) in the area around -1.45 V on the polarographic curve appears. The current intensity provided by a single-stranded DNA (peak III) is about 100 times greater than the intensity of peak II as an equal concentrated solution of native DNA. In the sonicated samples for less than 25 minutes there was evident no peak III (Fig. S2). This indicates that the sonication for 25 minutes leads to the denaturation effects on DNA. The sonication time for the preparation of DNA was 20 minutes, when no evidence of DNA denaturation after sonication was observed.

**A**

**B**

**Figure S2.** The polarographic curves: A) (1) DPP curves of ctDNA, (2) DPP curve of denaturated DNA, B) DPP curve of (1) DNA sonicated 10 min, (2) DNA sonicated 20 min, (3) DNA sonicated 25 min

**Naphthoquinones measured in the solid phase**

**Figure S3.** Raman spectra of all measured naphthoquinones in the solid phase.

**Summary results of all the measured SERS Raman spectra of DNA modified by selected naphthoquinones**

The SERS method was used to confirm the hypothesis that substances bind to DNA and to confirm which main bases are modified after this binding to DNA. We presume that DNA was in a double helix. From all measured spectra it is obvious there is decreasing the SERS intensity as a consequence of the binding of various compounds to the DNA (Fig. S4-S8). For the comparison of the SERS spectra measured, both native and denatured DNA were used. The native DNA provides several very well developed bands in the spectrum. By contrast, in the SERS spectrum of denatured DNA there are clearly visible the bands of individual bases([Gaudry and others 2006](#_ENREF_29); [Lee and others 2004](#_ENREF_47); [Wei and others 2006](#_ENREF_91)). In DNA modified of 1,4-naphthoquinone there are bands 1209 cm^-1^ and 1405 cm^-1^, which correspond to the vibrations of adenine and guanine. These bands are influenced by a local denaturing effect in DNA modified of 1,4-naphthoquinone.

1)

2)

**Figure S4.** SERS Raman spectra of: 1) colloid + 1,4naphthoquinone + DNA (multiplied 5×), 2) colloid +1,4 naphthoquinone

1)

2)

**Figure S5.** SERS Raman spectra of: 1) colloid + binaphthoquinone + DNA (multiplied 5×), 2) colloid + binaphthoquinone

1)

2)

**Figure S6**. SERS Raman spectra of: 1) colloid + juglone + DNA (multiplied 5×), 2) colloid + juglone

1)

2)

**Figure S7.** SERS Raman spectra of: 1) colloid + lawsone + DNA (multiplied 5×), 2) colloid + lawsone

1)

2)

**Figure S8.** SERS Raman spectra of: 1) colloid + plumbagin + DNA (multiplied 5×), 2) colloid + plumbagin

A characteristic feature for DNA modified by binaphthoquinone is a peak near 1152 cm^-1^, which corresponds to the native thymus DNA backbone vibrations. The spectrum of DNA measured modified by juglone has a major band at 1012 cm^-1^, which corresponds to the vibration of a native DNA backbone as well. The band 1396 cm^-1^ corresponds to the contribution of juglone itself. The spectra of DNA measured modified by lawsone and plumbagin are largely similar. A characteristic feature is a band at 1120 cm^-1^ (1073 cm^-1^) for them, which corresponds to the native thymus DNA backbone vibrations but this is certainly a contribution of this naphthoquinones themselves. It is obvious from the SERS spectra of all complexes measured that all naphthoquinones are bound to double-helical DNA and this represents a typical intercalating mode.
